# Modeling of In Vivo Electrochemical Noise: A Computational Framework to Optimize the Corrosion Monitoring of Biodegradable Magnesium Implants

**DOI:** 10.3390/jfb17050218

**Published:** 2026-05-02

**Authors:** Kirill Makrinsky, Alexey Klyuev, Oleg Batishchev

**Affiliations:** Frumkin Institute of Physical Chemistry and Electrochemistry, Russian Academy of Sciences, 31/4 Leninskiy pr., 119071 Moscow, Russia

**Keywords:** biodegradable magnesium implant, electrochemical noise, zero-resistance ammetry, coating degradation, implantable sensor, equivalent circuit modeling, Monte Carlo simulation, predictive experiment design, 3R principles

## Abstract

Biodegradable magnesium implants offer significant clinical promise, but their safe use requires reliable real-time in vivo monitoring of coating integrity. Existing methods lack sufficient sensitivity and temporal resolution to detect degradation at early stages, and there are no computational tools able to predict the success of a given sensor design before animal experiments. In the present paper, we present BioElectroSynth—a digital simulator of an implantable zero-resistance ammetry (ZRA) corrosion sensor in a mouse model. The simulator combines electrochemical noise, cardiac and muscular bioelectric interference, and instrumental limitations into a unified model, enabling virtual experiments, which mimic the complexity of the in vivo system. Using Monte Carlo analysis, we establish that a 2% breach in a chitosan coating on an AZ91 magnesium alloy electrode is statistically detectable from approximately 30 recordings of 30 s each, and quantify how electrode area, its location, sampling rate, and coating quality jointly determine detection sensitivity. The framework provides the first quantitative tool for predicting in vivo experiment feasibility from standard in vitro electrochemical data alone. By identifying instrument and design configurations that are statistically underpowered before any animal use, the approach directly supports the 3R principles of humane research.

## 1. Introduction

Biodegradable magnesium alloys represent an important class of orthopedic and cardiovascular implant materials, as their gradual resorption eliminates the need for secondary removal surgery [1,2]. For instance, commercially available alloys like AZ91 exhibit suitable mechanical properties and adequate corrosion resistance in physiological media, making them potential candidates for temporary fixation devices. However, the clinical application of Mg-based implants depends on predictable degradation kinetics: rapid corrosion generates hydrogen gas, increases local pH, and reduces mechanical integrity before tissue healing is complete [3,4].

Protective coatings such as biopolymer films based on chitosan [5], polylactic acid (PLA) [6], and hydroxyapatite (HAp) composites [7] are frequently used to delay early-stage corrosion. The physical integrity of these coatings strongly influences the implant’s overall performance, making in situ monitoring essential. However, capturing this degradation dynamically presents a significant practical challenge. Conventional imaging techniques lack real-time capabilities, while standard active electrochemical methods can artificially disturb the delicate coating/tissue interface [8,9].

To address these methodological limitations, electrochemical noise analysis (ENA) via zero-resistance ammetry (ZRA) offers a compelling instrumental solution. By recording spontaneous current fluctuations between two identical electrodes fabricated from the investigated implant material, ENA serves as an effective, non-destructive technique for corrosion monitoring [10,11]. Unlike conventional electrochemical impedance spectroscopy (EIS), which requires an external voltage or current perturbation, ZRA passively captures stochastic information about pitting, film breakdown, and hydrogen evolution events in real time [12,13].

Moving ZRA monitoring to the in vivo environment introduces complications absent from ordinary in vitro studies. The measured signal is a superposition of electrochemical noise, bioelectric potentials from cardiac (ECG) and muscular (EMG) activity, and instrumental noise [14]. In small animal models, the proximity of the femoral implant site to the heart and active muscles causes substantial bioelectric interference at the electrode pair. Furthermore, the strictly limited surface area available on a mouse implant (typically < 1.0 cm^2^) restricts the absolute amplitude of the corrosion signal, making it inherently difficult to isolate the target electrochemical data from the overwhelming bioelectric background. This motivates a computational approach that can generate each signal component independently and sweep the parameter space of electrode design and acquisition settings through simulation. To the best of our knowledge, no existing framework joins noise generation, anatomically parameterized bioelectric interference, frequency-dependent impedance filtering, and ADC simulation in a single model.

At present, no computational tools exist to predict whether a given sensor design can detect coating degradation in vivo before committing to animal experiments. Researchers must rely on trial-and-error, with each failed experiment consuming time, resources, and animal lives. A predictive framework would allow unfavorable designs to be identified and discarded computationally, in line with the 3R principles (Replacement, Reduction, Refinement) [15].

Three questions arise from the above-mentioned challenges. First, for what combinations of electrode area and coating quality is ZRA-based monitoring feasible at all, given that corrosion noise, ECG interference, and the instrumental floor each scale differently with area? Second, what is the smallest coating breach that can be reliably distinguished from the intact state, and how does this threshold depend on electrode design, sampling rate, and the number of recordings available? Third, which signal processing strategy extracts the most information from the noisy composite signal?

We address these questions using BioElectroSynth, a digital simulator of an implantable ZRA corrosion sensor in a mouse model. The simulator combines ten independently parameterized noise and interference sources, filtered through frequency-dependent equivalent circuits, so that realistic in vivo signals can be generated from standard in vitro electrochemical data.

## 2. Materials and Methods

### 2.1. Overview of the Signal Model

The current measured by a ZRA in the two-electrode configuration is modeled as:

(1) $$I_{measured}(t)=Q_{ADC}[I_{corr}^{loop}(t)+I_{bio}^{loop}(t)+I_{sensor}(t)]$$

where $I_{corr}^{loop}(t)$ is the corrosion-noise current flowing through the measurement loop, $I_{bio}^{loop}(t)$ is the current induced by bioelectric sources (ECG, EMG), $I_{sensor}(t)$ is the additive instrumental noise, and $Q_{ADC}[\cdot ]$ is the ADC quantization operator. Each component is described below.

**Figure 1:**
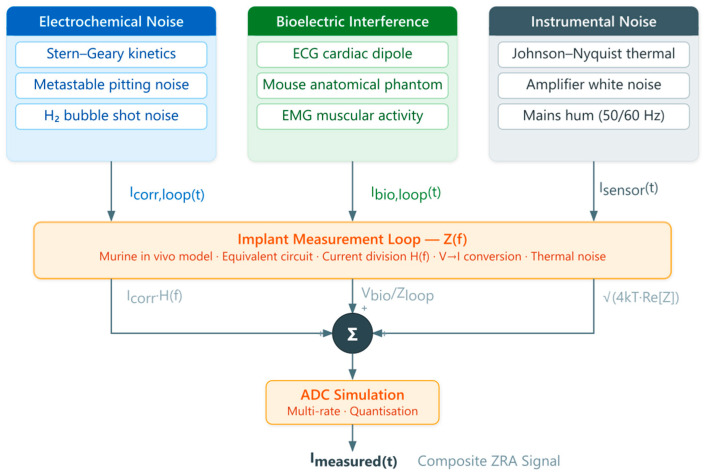
Block diagram of the BioElectroSynth signal generation pipeline.

Figure 1 presents the overall signal generation pipeline. Three independent source groups—electrochemical noise (corrosion current $I_{corr}$, comprising 1/*f*^2^ pitting noise, hydrogen bubble shot noise, and degradation trend), bioelectric interference (ECG from a mouse anatomical phantom and EMG bursts), and instrumental noise (Johnson-Nyquist thermal noise and amplifier white noise)—are generated at a high internal sampling rate (10 kHz). All current-generating components are filtered through the implant measurement loop impedance $Z_{loop}(\omega )$, which acts as a frequency-dependent current divider (Section 2.3). The filtered signals are summed and digitised by the ADC model, producing the final synthetic ZRA record $I_{measured}(t)$.

### 2.2. Electrochemical Noise Generation

The baseline corrosion current for every implanted electrode is calculated from standard EIS data for AZ91 in simulated body fluid (SBF) [16], using the Stern–Geary relationship [17]:

(2) $$I_{corr}=\frac{B\cdot A}{R_{ct}^{sp}}$$

where A [cm^2^] is the electrode area, $R_{ct}^{sp}$ [Ω·cm^2^] is the area-specific charge-transfer resistance, and B is the Stern–Geary coefficient determined from Tafel slopes (B≈0.03 V [18,19]).

The corrosion noise current comprises three physically distinct contributions. Metastable pitting noise arises from stochastic pit nucleation and repassivation events on the Mg surface, producing current fluctuations with a characteristic $1/f^{2}$ power spectral density (Brownian noise), consistent with experimental observations on corroding Mg alloys [12]. The pitting noise amplitude is scaled to approximately 10% of $I_{corr}$, consistent with the 1–10% range reported for metallic corrosion systems [10]. Hydrogen bubble shot noise originates from the discrete cathodic reaction ($2H_{2}O+2e^{-}\to H_{2}↑+2{OH}^{-}$), where each bubble detachment event produces a transient current pulse. The detachment rate is derived from Faraday’s law for the critical bubble volume ($r_{bub}\approx 20\;µm$), and individual pulses are smoothed by the RC time constant of the electrochemical double layer. A slow degradation trend can optionally be superimposed to simulate progressive coating loss.

The total electrode current is:

(3) $$I_{electrode}(t)=I_{corr}+I_{pit}(t)+I_{bub}(t)+I_{trend}(t)$$

### 2.3. Equivalent Circuit and Impedance Filtering

The electrochemical interface is represented by standard equivalent circuit elements like resistance (R), constant phase element (CPE), Warburg element (W), and inductance (L), combined into four topologies commonly used for AZ91 in simulated body fluid (SBF) [16]: single time constant (bare metal), two-time constants (coated surface), Warburg diffusion (extended exposure), and inductive loop (adsorbed intermediates). In the present Monte Carlo study, the two-time-constant topology was employed throughout, consisting of a film impedance ($R_{f}$, $CPE_{f}$) in series with the metal/electrolyte interface ($R_{ct}$, $CPE_{dl}$).

In the symmetric ZRA configuration, the total measurement loop impedance is:

(4) $$Z_{loop}(f)=R_{s}+2Z_{e}(f)+R_{shunt}$$

where $R_{s}$ is the electrolyte spreading resistance (estimated from tissue resistivity and electrode geometry), $Z_{e}$ is the electrode impedance, and $R_{shunt}$ is the ZRA input resistance. Each signal source sees a different transfer path through this circuit: corrosion noise is low-pass filtered by $Z_{e}/Z_{loop}$ (the double-layer capacitance shunts high-frequency fluctuations), bioelectric interference is converted from voltage to current via $1/Z_{loop}$ and instrumental noise is added directly. For the parameters used here ($R_{ct}^{sp}=300\;\Omega \cdot {\mathrm{cm}}^{2}$, $C_{dl}=25\;µF/{\mathrm{cm}}^{2}$), the cutoff frequencies are well above the signal bandwidth of the Monte Carlo study, so impedance filtering does not limit the corrosion noise signal.

### 2.4. Bioelectric Interference

The in vivo environment introduces bioelectric interference absent from benchtop measurements. The mouse body is represented by an anatomical phantom comprising nine oriented ellipsoids (torso, thorax, head, ears, limbs), providing a 3D geometry for electrode location.

Cardiac interference (ECG) is modeled using the standard current-dipole approach: the heart generates an electric field that propagates through the body tissues (modeled as a homogeneous volume conductor with conductivity σ=1.0 S/m), and the resulting potential difference between the two implant electrodes drives a current through the measurement loop impedance. The dipole magnitude is calibrated to reproduce surface ECG amplitudes of approximately 1.5 mV, consistent with murine electrophysiology [20]. The temporal ECG waveform is generated at 300–600 bpm using the NeuroKit2 library [21]. Electromyographic (EMG) interference is modeled as random bursts of broadband noise with a trapezoidal temporal envelope [22].

### 2.5. Instrumental Noise and ADC Simulation

Instrumental noise includes amplifier white noise, mains hum at 50/60 Hz, and Johnson-Nyquist thermal noise arising from the real part of the loop impedance. The composite signal is digitized using an ADC model that supports multiple resampling strategies (ideal sinc filtering, anti-aliasing decimation, and naive subsampling) with configurable quantization steps.

### 2.6. Coating Breach Detection: Monte Carlo Study Design

#### 2.6.1. Two-Zone Physical Model

To quantify the minimum detectable coating degradation on AZ91 implant, the electrode surface is divided into two zones: a coated zone of area A(1−θ) and a bare zone of area Aθ, where θ is the breach fraction. Both zones share the same metal/electrolyte interface parameters but differ in film coverage. The coated zone has an additional film impedance ($R_{f}^{sp}$, $CPE_{f}$), while the bare zone is in direct contact with the electrolyte. The two zones are electrically connected in parallel:

(5) $$Z_{electrode}=Z_{coated}\|Z_{bare}$$

Each zone generates independent corrosion noise scaled by its respective Stern-Geary current:

(6) $$I_{corr}^{\left(bare\right)}=\frac{B\cdot A\cdot \theta }{R_{ct}^{sp}},{\;I}_{corr}^{\left(coated\right)}=\frac{B\cdot A\cdot (1-\theta )}{R_{ct}^{sp}+R_{f}^{sp}}$$

**Figure 2:**
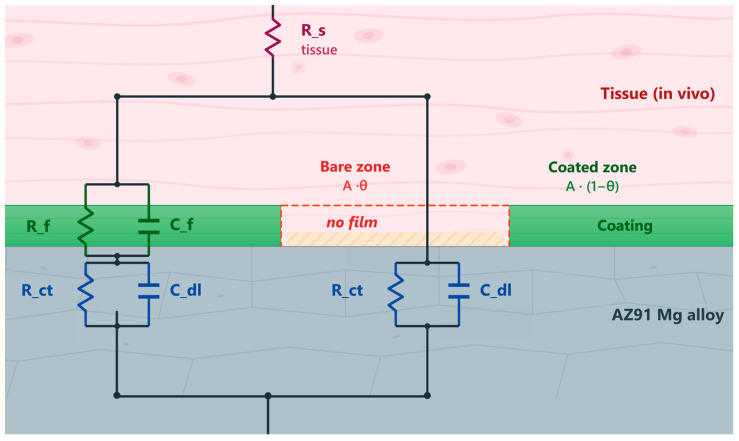
Two-zone coating breach model.

The current from each zone is filtered through zone-specific impedance transfer functions that reflect the parallel circuit topology. Figure 2 shows the physical cross-section of the two-zone electrode and the corresponding equivalent circuit. In the coated zone, current passes through the film impedance ($R_{f}\|C_{f}$) in series with the metal/electrolyte interface ($R_{ct}\|C_{dl}$), while in the bare zone, the coating is absent and the electrolyte contacts the metal surface directly, leaving only the interfacial impedance in the current path. Both zones share the tissue resistance $R_{s}$ in series. The breach fraction θ and total electrode area A are varied systematically across the Monte Carlo parameter space and fixed material parameters ($R_{f}$, $C_{f}$, $R_{ct}$, $C_{dl}$) are derived from in vitro EIS measurements.

#### 2.6.2. Material Parameters

The metal interface parameters correspond to AZ91 in SBF at early exposure: $R_{ct}^{sp}=300\;\Omega \cdot {\mathrm{cm}}^{2}$, $C_{dl}=25\;µF/{\mathrm{cm}}^{2}$ [16,19,23]. Coating parameters are: $R_{f}^{sp}=100$ Ω·cm^2^, $C_{f}=800$ µF/cm^2^. These values of $R_{f}^{sp}$ and $C_{f}$ were chosen to represent a worst-case scenario (a highly swollen/porous hydrogel-like state), making the coating highly transparent to alternating bioelectric fields. Instrumental noise parameters used: 0.05 µA RMS amplifier noise, 0.05 µA ADC step.

#### 2.6.3. Factorial Design

A total of 3300 Monte Carlo realizations (5 electrode areas × 11 breach fractions × 2 sampling rates × 30 seeds, 30 s each) were simulated using the two-zone impedance model with $R_{f}^{sp}=100\;\Omega \cdot {\mathrm{cm}}^{2}$. For each realization, four candidate detection metrics were computed: full RMS, moving-average RMS (1 s window), residual RMS after moving-average subtraction, and Chebyshev noise spectroscopy coefficients. An OLS regression of each metric against the breach fraction θ yielded the sensitivity slope $b_{1}$ and residual scatter σ; statistical detectability was assessed at significance level α=0.05. The parameter ranges used in the simulations are summarized in Table 1.

**Table 1:** Monte Carlo parameters.

Factor	Values	Rationale
Breach fraction θ	0, 0.01, 0.02, 0.05, 0.08, 0.10, 0.15, 0.20, 0.30, 0.40, 0.50	From intact film to 50% degradation
Electrode area A [cm^2^]	0.05, 0.10, 0.20, 0.50, 1.0	Practical implant size range
$\mathrm{Sampling}\;\mathrm{rate}\;f_{s}$ [Hz]	10, 100	Practical bandwidth range

#### 2.6.4. Signal Metrics and Statistical Analysis

Each realization is evaluated using four detection metrics. The first two are the standard root-mean-square (RMS) current and a 1 s moving-average RMS. The third metric is the residual RMS obtained after moving-average subtraction, which effectively isolates frequencies above 1 Hz. Finally, Chebyshev noise spectroscopy is applied by projecting the signal onto discrete Chebyshev polynomials, allowing us to extract power on the 0.4–2 s timescale characteristic of corrosion noise [24].

In addition to the detection metrics, power spectral density (PSD) estimates are computed using Welch’s method (Hann window, 50% segment overlap). Each segment is mean-subtracted before windowing (constant detrend); no additional linear or polynomial detrending is applied, as the synthetic signals are stationary within each recording by construction.

For each configuration, a linear regression of metric values against breach fraction θ is fitted across all Monte Carlo realizations, yielding a slope $b_{1}$ (sensitivity) and residual standard deviation σ.

The minimum detectable breach fraction can be derived using a standard two-sample hypothesis test comparing *N* independent measurements against *N* intact baseline recordings. Based on the Student t-distribution, the minimum detectable degradation is given by:

(7) $$\theta_{det}(N)=\frac{t_{\alpha /2,\nu }\cdot \sigma }{b_{1}}\cdot \sqrt{\frac{2}{N}}$$

where $t_{\alpha /2,\nu }$ is the critical *t*-value for significance level α and ν degrees of freedom.

This derivation highlights a crucial parameter: the ratio $\sigma /b_{1}$, which we define as the noise-to-sensitivity ratio (NSR). The NSR is a dimensionless figure of merit that remains independent of the number of recordings *N*. Equation (7) highlights that the detection threshold decreases with the square root of the number of observations, following the central limit theorem.

#### 2.6.5. Operating Window Bounded by ECG, Area, and Film Resistance

To quantify the optimal working window for in vivo ZRA monitoring, a Monte Carlo simulation was performed using the same two-zone impedance model and noise chain employed in the breach detection study. For each combination of electrode area A and area-specific coating resistance $R_{f}^{sp}$, 15–30 independent stochastic realizations (10 s, $f_{s}^{int}$ = 10 kHz) were generated, and the RMS of the measured corrosion current was compared against the ECG-induced current and the amplifier noise floor (0.05 µA). Electromyographic interference was deliberately excluded from this comparison because the optimization was performed for the case of a fully restrained animal (e.g., under anesthesia), where muscular activity is effectively absent. Since the cardiac rhythm cannot be suppressed by mechanical restraint, ECG constitutes an irreducible bioelectric background under such conditions.

### 2.7. Software Implementation

**Figure 3:**
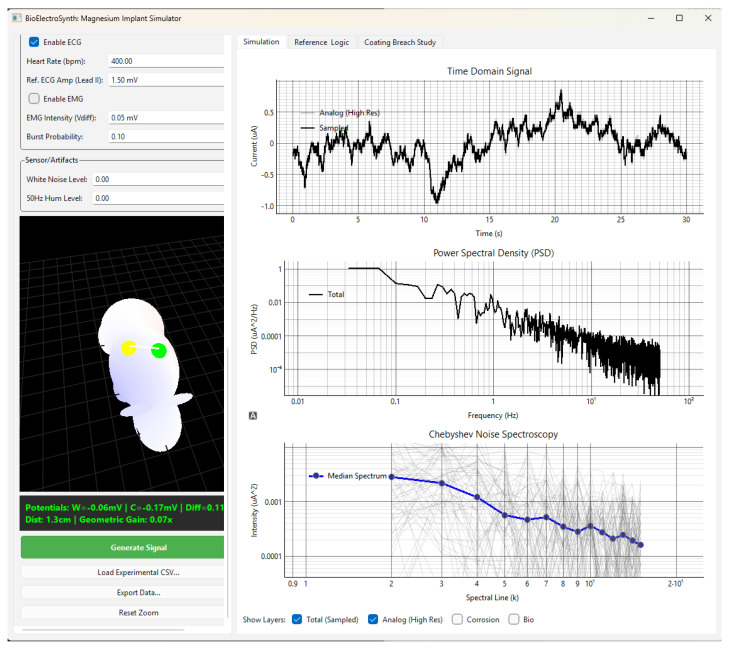
Screenshot of the BioElectroSynth interface. Left: 3D mouse phantom with working (yellow) and counter (green) electrode markers, ECG/EMG controls, and real-time electrode potentials. Right: time-domain ZRA signal, PSD, and Chebyshev noise spectroscopy (blue—median; grey—individual realisations).

The simulator is implemented in Python 3.12 using NumPy 2.2.1, SciPy 1.16.3 (signal processing), NeuroKit2 0.2.12 (physiological waveform generation), and PyQt6 6.10.2 (graphical interface). The interactive GUI (Figure 3) includes a 3D anatomical mouse phantom for electrode placement, real-time signal generation, power spectral density estimation, and Chebyshev noise spectroscopy, so that the full parameter space can be explored without writing code. Equivalent circuits are implemented as composable objects supporting arbitrary nesting of series and parallel combinations. All user-adjustable parameters are restricted to physically meaningful positive ranges (e.g., electrode area > 0, charge-transfer resistance > 0) and the graphical interface validates inputs before simulation to prevent numerical instability. All source code is available at GitHub repository https://github.com/KMakrinsky/bioelectrosynth (accessed on 1 April 2026).

## 3. Results

### 3.1. Signal Component Separation (Model Validation)

**Figure 4:**
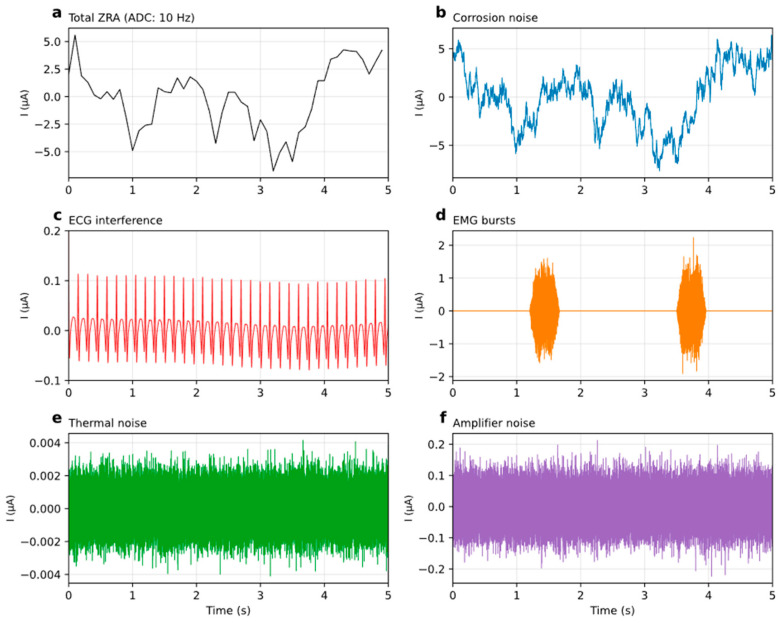
Decomposition of a synthetic ZRA signal into six components. (**a**) Total measured current after digitization by an ideal 10 Hz ADC ($\Delta_{LSB}=0.05$ µA). (**b**) Corrosion noise (**c**) ECG interference. (**d**) EMG burst interference. (**e**) Johnson-Nyquist thermal noise. (**f**) Amplifier white noise.

Figure 4 shows a synthetic ZRA signal decomposed into six components. Parameters: $A=0.50\;{\mathrm{cm}}^{2}$, $R_{ct}^{sp}=300\;\Omega \cdot {\mathrm{cm}}^{2}$, breach θ=0.1, heart rate 400 bpm, EMG burst rate 0.3 s^−1^. The upper panels compare the signal as seen by the instrument with the underlying corrosion process: Figure 4a is the total current after digitization by a 10 Hz ADC ($\Delta_{LSB}=0.05$ µA), while Figure 4b is the corrosion noise at full 10 kHz bandwidth, where 1/*f*^2^ random-walk fluctuations and hydrogen bubble transients are visible but are smoothed out by the anti-aliasing filter at 10 Hz. ECG interference (Figure 4c, ~0.1 µA peak-to-peak at geometric gain 0.15) has the periodic structure of the mouse cardiac cycle, with R-peaks at ~6.7 Hz. EMG bursts (Figure 4d) appear as sporadic broadband current transients with amplitudes comparable to ECG. Johnson–Nyquist thermal noise (Figure 4e, ~0.1 nA RMS) and amplifier white noise (Figure 4f, ~0.01 µA RMS) are both negligible with these electrode parameters. Each component can be toggled independently in the GUI, allowing the user to see how each noise source affects detection.

### 3.2. Electrode Area and Coating Resistance Optimization

**Figure 5:**
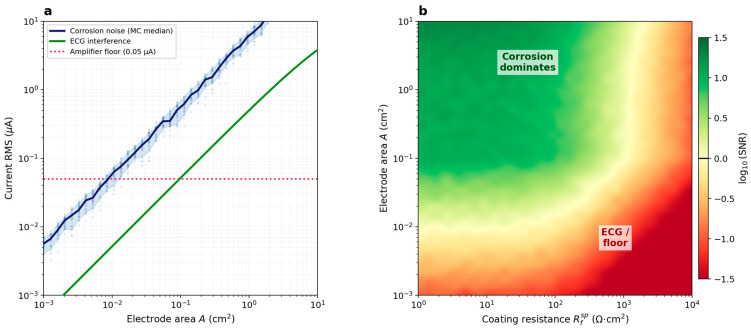
Monte Carlo electrode area optimization (bare AZ91, mouse chest). (**a**) Corrosion noise RMS vs. electrode area: individual MC realizations (pale blue dots), 10th–90th percentile band, and median (dark blue); ECG-induced current (green); amplifier floor (red dotted). (**b**) SNR phase diagram: as a function of coating resistance $R_{f}^{sp}$ and electrode area A.

Figure 5a shows the bare-metal reference case. Both the corrosion noise RMS (MC median, blue) and the ECG interference (green) scale proportionally with the electrode area. For bare AZ91, the corrosion signal remains consistently above the ECG interference across the entire simulated range. However, at the lower end, the constant amplifier noise floor (0.05 µA) limits detectability below *A* ~ 0.01 cm^2^. The resulting working window for uncoated AZ91 spans approximately three decades of electrode area.

Figure 5b extends this to coated electrodes as a two-dimensional phase diagram. The color map shows ${log}_{10}$(SNR), defined as the ratio of median corrosion noise RMS to the dominant interference source (ECG or amplifier floor, whichever is larger), as a function of $R_{f}^{sp}$ and A. The green region (SNR > 1) marks configurations where corrosion noise exceeds all interference. This diagram serves as a practical design map: for a given coating resistance and electrode area, one can immediately determine whether ZRA monitoring is feasible. The most important trend visible in Figure 5b is the steep shrinkage of the green region as $R_{f}^{sp}$ increases—a direct consequence of the coating suppressing corrosion current while the ECG background remains unaffected by it. A separate, area-driven limit appears at the bottom of the map: below *A* ~ 0.01 cm^2^, the corrosion signal of even a bare, uncoated electrode does not reach the amplifier noise floor.

### 3.3. Monte Carlo Study: Coating Breach Detection

**Figure 6:**
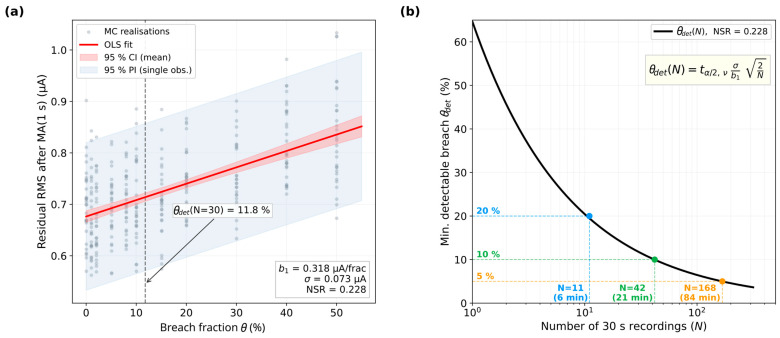
Monte Carlo coating breach detection (3300 realizations, 30 s recordings, residual RMS metric, $R_{f}^{sp}=100$ Ω·cm^2^). (**a**) Representative OLS regression at A=0.5 cm^2^, $f_{s}=10$ Hz. (**b**) Detection threshold curve for the same configuration (two-sample *t*-test at α=0.05).

The representative configuration (A=0.5 cm^2^, $f_{s}=10$ Hz, $R_{f}^{sp}=100\;\Omega \cdot {\mathrm{cm}}^{2}$) is shown in Figure 6a. The OLS fit yields *b*_1_ = 0.318 and *σ* = 0.073, giving NSR = 0.228. Applying Equation (7) to this configuration (Figure 6b), a 20% breach is detectable from 11 recordings (6 min), a 10% breach from 42 recordings (21 min), and a 5% breach from about 168 recordings (84 min).

Data for all 10 electrode configurations and four detection metrics is given in Table 2. The residual RMS gives the lowest threshold at A≥0.2 cm^2^ (7–23%): subtracting the moving average removes the most variable low-frequency 1/*f*^2^ components without proportionally reducing the sensitivity slope. At the smallest area (A=0.05 cm^2^), the residual and full RMS perform comparably. The NSR spans from 0.14 (*A* = 1.0 cm^2^, *f_s_* = 100 Hz) to 0.55 (*A* = 0.05 cm^2^, *f_s_* = 10 Hz), confirming that the detection curve must be evaluated for each electrode design separately.

**Figure 7:**
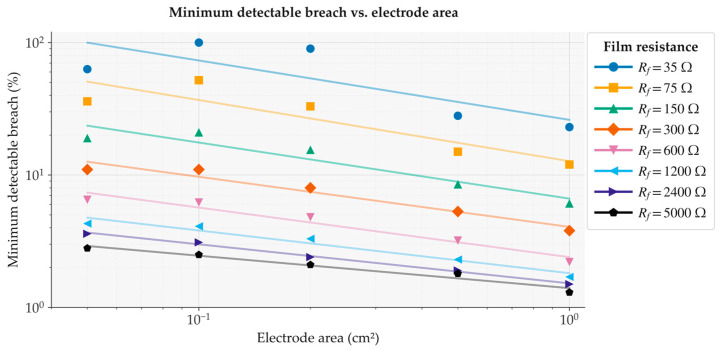
Minimum detectable breach fraction (%) as a function of electrode area.

How much the threshold matters in practice depends on the coating quality. Figure 7 sweeps $R_{f}^{sp}$ from 35 to 5000 Ω·cm^2^ at five electrode areas using the residual RMS metric and *N* = 30 recordings. For high-quality coatings above 1000 Ω·cm^2^, the impedance contrast between intact and breached zones is large enough that a 2–4% breach is detectable even at 0.05 cm^2^ electrodes. At the baseline value used throughout this study ($R_{f}^{sp}=100\;\Omega \cdot {\mathrm{cm}}^{2}$), the threshold at the same area is approximately 25%, which reflects the deliberately weak impedance contrast of this worst-case scenario. In all cases, larger electrodes lower the threshold by increasing the total corrosion current collected.

## 4. Discussion

### 4.1. Methodological Context and Comparison with Existing Approaches

Monitoring the in vivo degradation of biodegradable magnesium implants has remained an unsolved instrumentation problem for decades. All methods used in practice (micro-CT, radiography, hydrogen collection, serum ion measurements) share a common limitation: none of them is continuous, passive, and directly sensitive to the electrochemical state of the implant surface.

Electrochemical methods have been applied to implanted metallic electrodes in living animals since the 1960s. Colangelo et al. used linear polarization with a transcutaneous needle probe to measure corrosion rates of steel, cobalt and molybdenum implanted in dog muscle [25], and Nakayama et al. later performed full anodic polarization scans on a compact three-electrode assembly implanted in rabbit muscle [26]. These pioneering studies demonstrated that active electrochemical measurements in vivo are technically feasible for passive alloys. Neither approach, however, is transferable to biodegradable magnesium alloys. Unlike stainless steel or titanium, Mg does not form a stable passive film at its natural rest potential, so any anodic perturbation (whether the large potential sweep of potentiodynamic polarization or the small increments of linear polarization) causes irreversible dissolution of the implant material rather than probing the integrity of a protective layer. EIS shares the same limitation: it requires an externally imposed sinusoidal perturbation and is impractical in a freely moving animal.

The indirect methods are not better in terms of temporal resolution. Micro-CT resolves volumetric loss above roughly 5% but requires repeated anesthesia and imaging sessions spaced days to weeks apart [27,28]. Transdermal, or even implantable, hydrogen sensors detect the corrosion by-product rather than the process itself, and H_2_ exchanges rapidly with surrounding tissue, making early-stage quantification unreliable [29,30,31]. Serum Mg^2+^ measured by ICP [30] reflects systemic ion release but cannot localize degradation to a single implant, and the physiological background of 0.7–1.0 mM [32] masks small increments. None of these methods can detect a localized breach in a protective coating before it progresses to bulk degradation.

ZRA-ENA occupies the gap left by all these approaches. It is passive (no external perturbation is applied) continuous, and directly sensitive to the stochastic electrochemical noise generated at the corroding metal surface. The distinguishing feature relevant to coating monitoring is that ZRA-ENA is specifically sensitive to the impedance contrast between intact and breached zones: a small bare patch on an otherwise coated electrode generates a large noise current relative to the intact surface, making early breach detection possible in principle. Methods that integrate over the whole implant surface, such as weight loss, ICP, micro-CT, are blind to this contrast by construction.

What has been missing is a quantitative framework for translating this principle into a practical sensor design: how large must the electrode be, how protective the coating must be, and how many recordings are needed before a breach of a given size can be statistically confirmed? The present study addressed these questions through an electrochemistry-based Monte Carlo simulation that models each signal and interference source explicitly, producing detection thresholds as a function of the hardware parameters that an experimenter can control.

### 4.2. The Electrode Area and Coating Resistance Trade-Off

The area optimization (Figure 5) shows a two-sided design trade-off that, to our knowledge, has not been quantified in the Mg implant literature. Electrodes that are too small produce corrosion current noise below the instrumental floor; electrodes that have too large ohmic resistance of protective coating let ECG interference dominate. For bare AZ91, the working window between these two limits spans nearly three decades of electrode area.

The SNR phase diagram (Figure 5b) shows this trade-off directly. The green (SNR > 1) region narrows with increasing coating resistance. The lower boundary is set by the amplifier noise floor and demands an electrode area of at least ≈0.01 cm^2^. As $R_{f}^{sp}$ rises, the corrosion current is progressively suppressed while the ECG interference remains unchanged, and the coating resistance itself imposes an upper limit. Beyond $R_{f}^{sp}\approx 10^{3}\;\Omega \cdot {\mathrm{cm}}^{2}$ the corrosion signal falls below the ECG + Noise background at any electrode area, and the green region disappears. In practice, this means that for bare or lightly coated electrodes the working window is broad and electrode area is not critical, whereas for well-coated implants instrument sensitivity becomes the bottleneck, and low noise potentiostats with sub-µA resolution are needed.

These results can be evaluated against actual implant geometries. For example, MAGNEZIX^®^ compression screws (Syntellix AG) have shaft diameters of few millimeters and lengths of 10–40 mm, yielding exposed surface areas of approximately 0.5–5 cm^2^ depending on the selected size. Most orthopedic Mg implants therefore fall within the working window for bare or moderately coated alloys (Figure 5a). Clinical Mg alloys other than AZ91 (WE43, LAE442, MgYREZr) have comparable or lower corrosion rates, so the phase diagram shifts along the current axis but retains its shape; the design rules derived here are transferable in form, though the specific boundaries depend on B and $R_{ct}^{sp}$. Moreover, $R_{f}$ is not static: in vivo, polymer coatings hydrate, swell, and eventually delaminate, so the operating window drifts leftward across the phase diagram over implantation time. The sensor must remain within the working window throughout the monitoring period.

### 4.3. Early-Stage Degradation Kinetics and Biomechanical Significance of Localized Breach Detection

The degradation of a magnesium implant does not proceed at a constant rate. Upon first contact with physiological fluid, the bare metal surface undergoes rapid anodic dissolution and hydrogen evolution; the nascent Mg(OH)_2_ layer provides only partial protection, and its conversion to soluble MgCl_2_ by chloride ions sustains aggressive attack until a stable mixed-phase corrosion product layer gradually covers the surface [33]. Electrochemical studies under flow conditions confirm that the charge-transfer resistance *R_ct_* is at its lowest in the first hour of immersion and rises to a plateau only after the initial passivation film breaks down and reforms [34]. Ion-release measurements on WE43 alloy show a parallel pattern: Mg^2+^ release rate is highest in the first 30 min, then falls sharply, and reaches a low, quasi-steady value only after 168 h as corrosion products cover the entire surface [35]. It is precisely during this transient window that a localized breach in a protective coating is most consequential: the bare patch is exposed at the moment of peak corrosion driving force, before any passivating mineral layer can form.

The biomechanical consequences of a breach protective at this stage are disproportionate to its area. van Gaalen et al. demonstrated that the minimum cross-sectional area at the deepest pitting defect is the dominant predictor of residual tensile strength in a rare-earth Mg alloy, and that this relationship holds independently of how pitting is distributed across the implant surface [36]. At electrode areas of 1.0 cm^2^ with a coating resistance of 1200 Ω·cm^2^, our framework resolves a minimum detectable breach of approximately 2% from *N* = 30 recordings (Figure 7), corresponding to 0.02 cm^2^ of bare metal. Defects of this scale are mechanically consequential well before they produce any measurable change in implant geometry or mass, which is precisely the regime where cross-sectional area loss, rather than bulk degradation, governs mechanical failure.

For in vivo coating research, this sensitivity threshold opens an experimental question that has been inaccessible so far: at what point after implantation does a given coating first fail, and how does that timing correlate with subsequent mechanical and histological outcomes measured at explantation? Current animal studies characterize coating performance through terminal time points (typically 4, 8, and 12 weeks) and infer degradation kinetics retrospectively from micro-CT or weight loss. Continuous ZRA logging would replace this discrete sampling with a time-resolved record of the actual breach event, allowing direct comparison of coating formulations within the same cohort and reducing the number of animals needed to establish statistically meaningful degradation timelines.

### 4.4. Limitations and Future Work

Several modeling choices limit how broadly the results can be interpreted. The anatomical phantom approximates the mouse body as nine homogeneous ellipsoids, whereas real tissue has spatially varying conductivity and anisotropic muscle architecture. This will shift the absolute amplitude of ECG interference at the electrode pair, but the qualitative structure of the phase diagram in Figure 5b should be robust to these deviations. Bioelectric parameters were taken from published mouse electrophysiology rather than measured in the target implant configuration; individual variability in heart rate, posture, and locomotor activity during free movement will widen the interference distributions beyond those simulated here.

On the electrochemical side, the charge-transfer resistance, double-layer capacitance, and coating impedance were fixed at values derived from in vitro EIS data on AZ91 in simulated body fluid. These parameters change substantially in vivo as proteins adsorb, inflammatory cells accumulate, and corrosion products mineralize over the first weeks of implantation. The simulator currently treats them as time-invariant; allowing them to evolve according to measured in vivo trajectories is the most important planned extension. The Monte Carlo study swept coating resistance across nearly two orders of magnitude (Figure 7), but all other material and geometric parameters were held constant. Researchers working with other alloys or coating chemistries can substitute their own EIS-derived parameters and select from the four equivalent circuit topologies implemented in the GUI to re-run the Monte Carlo analysis for their specific system without modifying the source code. Extending the factorial design to include alloy composition, electrolyte formulation, and implant geometry would further broaden the applicability of the resulting design maps, and the modular architecture of BioElectroSynth is intended to support exactly this kind of incremental refinement as new experimental data become available.

All reported thresholds, NSR values, and working windows are model outputs. They are predictions about what a real sensor would measure, not measurements themselves. Experimental validation using ZRA recordings from freely moving implanted animals, with the electrode geometries and sampling protocols identified as adequately powered here, is the necessary next step. Those experiments are in progress and will be reported in our next publication.

## 5. Conclusions

BioElectroSynth provides the first physics-based framework for predicting whether a ZRA corrosion sensor can detect surface degradation on a biodegradable magnesium implant before any animal experiment is conducted. The simulator translates standard in vitro EIS data, routinely collected during biomaterial characterization, into quantitative predictions of in vivo detectability, closing a gap that has so far forced researchers to discover sensor failures only after committing to animal studies.

The noise-to-sensitivity ratio introduced here gives biomaterials researchers a concrete screening criterion applicable across the full range of implant surface states, from bare alloy to fully coated electrode. For uncoated Mg alloys, the working window spans nearly three decades of electrode area, meaning that the corrosion signal of the alloy itself is accessible over the entire practical size range of orthopedic implants. For coated surfaces, the framework quantifies how coating resistance and electrode area jointly determine detectability: high-barrier coatings with R_f above 1000 Ω·cm^2^ allow breach fractions as small as 2% to be resolved from 30 recordings of 30 s, a sensitivity that corresponds to the spatial scale at which localized pitting begins to govern residual mechanical strength. This makes it possible to compare not only coating formulations with each other, but also coated and uncoated conditions within the same experimental design.

The framework is not limited to AZ91. Researchers working with other Mg alloys, surface treatments, or equivalent circuit topologies can substitute their own electrochemical parameters into BioElectroSynth and obtain design maps specific to their system. As the library of characterized biodegradable metal surfaces grows, the approach provides a reusable computational substrate for translating electrochemical bench data into experimentally actionable sensor specifications for identifying underpowered configurations before any animal lives are used.

## Figures and Tables

**Table 2:** Detection thresholds for different metrics and configurations ($R_{f}^{sp}=100\;\Omega \cdot {\mathrm{cm}}^{2}$). Bold indicates the best (lowest) threshold for each configuration.

A [cm^2^]	$f_{s}$ [Hz]	RMS	Resid (1 s)	MA (1 s)	Cheb (2–5)
0.05	10	37.7	**28.2**	41.8	69.5
	100	33.4	**31.9**	36.1	47.8
0.10	10	**34.6**	37.0	36.5	60.8
	100	51.5	**18.5**	64.7	>100
0.20	10	48.8	**22.6**	55.1	64.6
	100	34.5	**21.1**	39.6	67.1
0.50	10	40.6	**11.8**	53.2	>100
	100	29.2	**10.7**	36.3	47.8
1.00	10	30.5	**9.1**	40.2	>100
	100	21.1	**7.4**	26.4	33.2

## Data Availability

The original data presented in the study are openly available in https://github.com/KMakrinsky/bioelectrosynth (accessed on 1 April 2026).

## Abbreviations

The following abbreviations are used in this manuscript:

ZRA	Zero-Resistance Ammetry
ENA	Electrochemical Noise Analysis
EIS	Electrochemical Impedance Spectroscopy
